# Lateral violence and turnover ideation in nurses: multiple mediating roles of coping styles and psychological resilience

**DOI:** 10.3389/fpubh.2026.1774116

**Published:** 2026-03-05

**Authors:** Mengjie Sun, Xueping Cui, Miaomiao Li, Xuehua Chen, Aili Lou, Yuecai Zhang, Huiping Zhao, Ke Zhang, Wenjing Zhi, Rui Li, Yanmin Shao

**Affiliations:** Zhoukou Central Hospital, Zhoukou, Henan, China

**Keywords:** coping styles, lateral violence, mediating effects, multiple mediation model, nurses, psychological resilience, turnover ideation

## Abstract

**Objective:**

To explore the relationship between nurses’ Lateral violence and turnover intentions, and the chain-mediating effects of psychological resilience and coping styles.

**Methods:**

A total of 281 nurses were recruited in this cross-sectional study. The questionnaire consisted of sections on lateral violence, psychological resilience, coping styles and turnover intentions. Descriptive correlation studies examined the relationships among the research variables, while structural equation modeling tested the validity of the proposed theoretical framework.

**Results:**

Lateral violence was positively correlated with turnover intention (*r* = 0.517, *p* < 0.05) and negative coping (*r* = 0.445, *p* < 0.01), and was negatively associated with psychological resilience (*r* = −0.319, *p* < 0.05) and positive coping (*r* = −0.272, *p* < 0.05). The effect of psychological resilience on turnover intentions was partially mediated by negative coping. Psychological resilience and negative coping styles formed a concurrent and sequential chain-mediating role between lateral violence and turnover intention.

**Conclusion:**

These findings reveal significant associations among lateral violence, psychological resilience, coping styles, and turnover intention. The structural equation model highlights the explanatory role and mediating effects of psychological resilience and coping strategies in the relationship between lateral violence and turnover intention. It is suggested that assessing nurses’ psychological resilience and reducing negative coping strategies could be considered as potential approaches within health management to address the observed link between lateral violence and turnover intention.

## Introduction

Lateral violence (LV), also known as horizontal violence (HV), refers to overt or covert workplace hostility within the same organization or group, manifesting as ridicule, verbal abuse, isolation, sabotage, scapegoating, and other forms (1). As a form of interpersonal conflict, nurses have increasingly become the healthcare professionals most affected by lateral violence within healthcare systems, drawing attention from global nursing organizations (2, 3). A recent systematic review revealed that the incidence of lateral violence among nurse ranges from 7 to 83%, with an overall prevalence rate of 33.08% (4). Notably, given the high prevalence of this violence, attention must be paid to its impacts and consequences. Physical and emotional manifestations such as insomnia, chronic fatigue, anxiety, depression, fear, occupational burnout, and post-traumatic stress disorder syndrome progressively emerge among nurses (2, 3, 5). When these physical symptoms and negative emotions exceed nurses’ tolerance levels, they can drive the intention to leave their jobs in search of relief (6).

Turnover intention refers to an employee’s deliberate inclination to leave an organization and seek alternative employment opportunities, serving as a precursor to actual resignation (7). Previous studies have confirmed that lateral violence correlates with turnover intention and constitutes a significant predictor thereof (8, 9). A Chinese study revealed that the incidence of lateral violence among nurses reached 56.6%, with 21.67% of these nurses exhibiting turnover intention (10). Recent reports indicate a global average nurse turnover rate of 16% (11). Frequent staff turnover not only reduces organizational efficiency and increases hospital labor costs, exacerbating nursing shortages, but also compromises the continuity and quality of patient care services (12). Therefore, mitigating the impact of nurses’ lateral violence on turnover intention holds significant implications for patient safety and the development of the international nursing workforce.

To further explore the underlying mechanisms between lateral violence and turnover intention, this study is grounded in the Conservation of Resources Theory and the Stress and Coping Theory. It positions psychological resilience and coping styles as key individual psychological factors influencing lateral violence, turnover intention, and mental health (13–15). Psychological resilience is defined as an internal resource, referring to a dynamic process through which individuals adapt well when facing various stressors (16, 17). This process is influenced by multiple factors, including social, psychological, and cultural dimensions. Psychological resilience is widely regarded as a protective factor for nurses’ career development. Existing evidence indicates that resilience can mitigate the negative impacts of workplace psychological violence, promote mental health (18), and is associated with lower turnover intention (13, 19). Coping styles refer to the cognitive and behavioral strategies individuals employ to alleviate stress. They primarily include problem-focused, constructive positive coping (such as accumulating resources or seeking organizational support to solve problems) and emotion-focused or avoidant negative coping (such as reducing distress through self-blame, avoidance, or denial). Coping styles are not static. Individuals may shift between positive and negative strategies depending on situational changes. Qualitative research similarly notes that nurses adopt different coping strategies when experiencing lateral violence, for example, seeking social support, actively adapting, remaining silent, avoiding conflict, or exhibiting complex reactions with intertwined emotions (20, 21). Previous research on lateral violence and coping strategies has shown an association between workplace violence encountered by nurses and their coping styles, with a negative correlation to positive coping and a positive correlation to negative coping (22). However, most existing studies have focused on indicators related to patient mental health (15), paying less attention to turnover intention as an outcome variable. Given that turnover intention is closely related to individual coping styles and that coping styles can significantly predict turnover intention (23), further investigation into this relationship is warranted.

Psychological resilience represents a broader concept encompassing an individual’s overall capacity to withstand and recover from stress, while coping styles refer to the specific methods employed during this process. These two constructs overlap and follow a logical progression, as psychological resilience can be viewed as the foundation that influences both the selection and implementation of coping strategies (24). Individuals with higher resilience likely possess greater internal resources, making them more inclined to adopt positive coping strategies (25–27). Conversely, depletion of these resources may increase one’s reliance on negative coping strategies. In theoretical terms, lateral violence constitutes a complex, stressful environment, psychological resilience serves as a positive internal resource, coping styles act as cognitive and behavioral strategies, and turnover intention represents the outcome of an individual’s internal deliberation. This leads to a proposed chained mediation pathway: lateral violence may erode psychological resilience, which in turn constrains and shapes an individual’s choice of coping style, ultimately influencing their turnover intention.

Existing literature has recognized the association between lateral violence and turnover intention, as well as the roles of psychological resilience and coping styles as independent risk or protective factors. However, research remains insufficient to place these psychological variables within a unified framework to examine whether they function in a temporally sequential, chained manner during the stress transmission process. Particularly within the specific high-pressure context of nursing work, where lateral violence acts as a chronic interpersonal stressor, its impact depletes individuals’ internal psychological resources (such as resilience), subsequently shapes their observable coping behaviors, and ultimately drives the decision to leave. This complete “resource → strategy → outcome” pathway still awaits empirical testing. Examining this sequential model, rather than only testing parallel mediation, can help clarify the temporal order and interactive relationship between “resource depletion” and “strategy selection” in the stress transmission process, thereby deepening our understanding of nurses’ psychological and behavioral response patterns under complex interpersonal stress.

**Figure 1:**
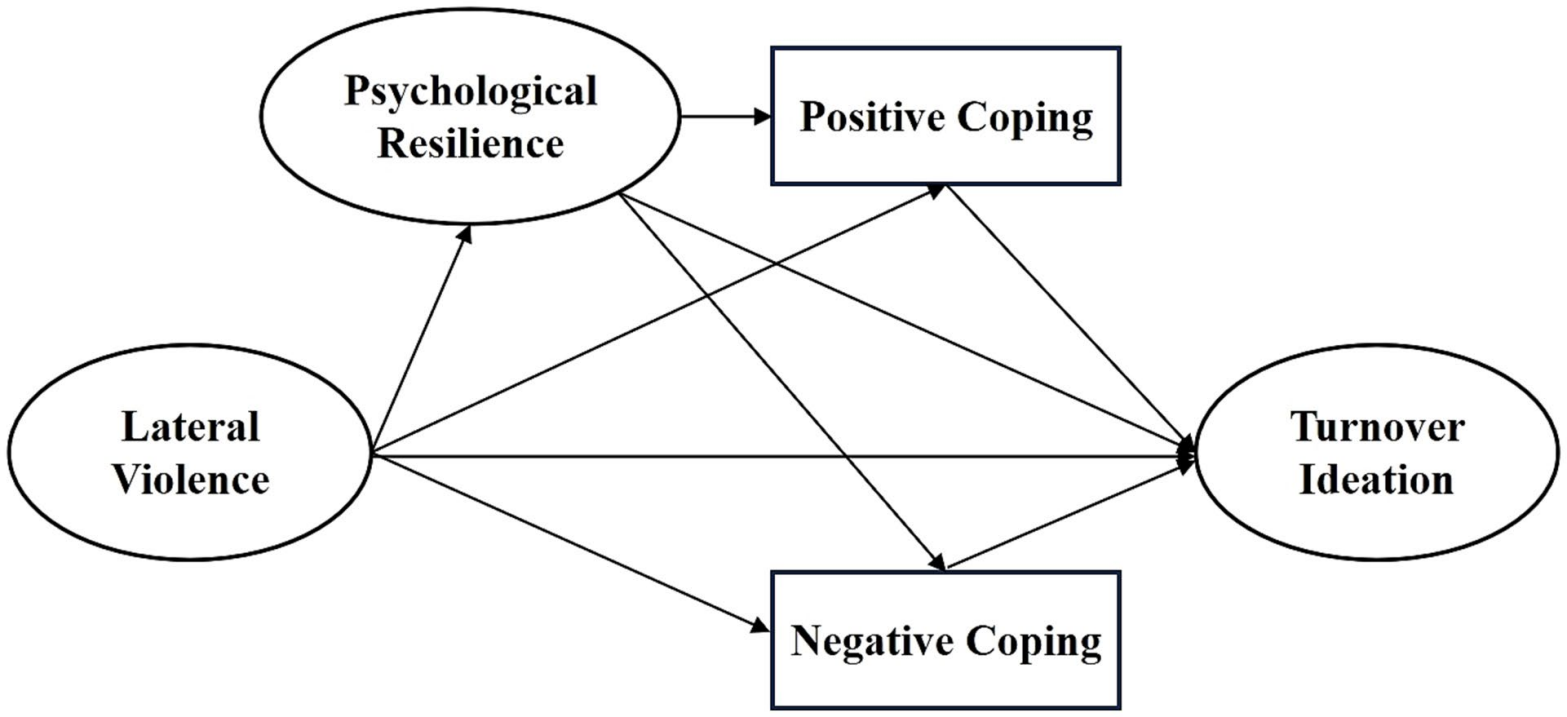
Theoretical model.

The following hypotheses are proposed (as illustrated in Figure 1, Theoretical Model):

*Hypothesis 1*: Lateral violence, psychological resilience, and coping styles have a direct effect on turnover intention.

*Hypothesis 2*: Psychological resilience and coping styles each play a mediating role between lateral violence and turnover intention.

*Hypothesis 3*: Psychological resilience and coping styles serve as chained mediators between lateral violence and turnover intention.

## Methods

### Study design

This study was a cross-sectional survey, adhering to the EQUATOR guidelines and the STROBE reporting methods, aiming to analyze the chain-mediated roles of psychological resilience and coping styles between lateral violence and turnover intention, rather than exploring causal relationships between variables.

### Participants and settings

This study employed a convenience sampling method, selecting clinical nurses from a tertiary grade-A hospital in Henan Province in October 2023. Inclusion criteria were as follows: (1) registered nurse; (2) at least one year of nursing work experience; (3) voluntary participation. Exclusion criteria included: nurses rehired after retirement, visiting nurses, and those not on duty during the survey period due to maternity leave, sick leave, studying abroad, etc.

The sample size was estimated using Kendall’s rough estimation method, requiring 10 to 15 times the number of questionnaire variables. With 19 variables in this study and accounting for a 20% rate of invalid questionnaires, the required sample size ranged from 228 to 342. This meets the general requirements for structural equation modeling, which typically suggests a sample size between 200 and 500. Data were collected anonymously via the “Questionnaire Star” application. A total of 281 valid questionnaires were ultimately collected for this study.

### Questionnaires

#### Demographic characteristics

The nine demographic information of the nurses was collected, including sex, age, work experience, marital status, educational background, professional qualifications, hospital employment type, monthly income, and department.

#### Lateral violence scale

The Lateral Violence Scale developed by Li et al. (28), was used to evaluate the frequency of lateral violence experienced by clinical nurses in the past 6 months. The scale consists of two dimensions: overt lateral violence (8 items) and covert lateral violence (11 items), totaling 19 items. It employs a Likert 5-point scoring system, ranging from “never” to “almost daily,” with scores assigned from 1 to 5. Higher scores indicate a higher frequency of lateral violence behaviors. The scale demonstrated good internal consistency, with a Cronbach’s α coefficient of 0.95 and a split-half reliability of 0.93. In this study, the overall Cronbach’s α coefficient for the scale was 0.980, while the coefficients for the two dimensions were 0.959 and 0.964, respectively.

#### Connor–Davidson resilience scale (CD-RISC)

Psychological resilience was assessed using the Connor–Davidson Resilience Scale (CD-RISC) (29). The scale consists of three dimensions: Tenacity (13 items), Strength (8 items), and Optimism (4 items), totaling 25 items. A 5-point Likert scale was employed, with response options ranging from 0 to 4: “Never” to “Always.” Scores were calculated on a percentage basis, with higher scores indicating stronger resilience in nurses when facing difficulties and challenges. In this study, the overall Cronbach’s α coefficient for the scale was 0.965, with dimension-specific coefficients of 0.946, 0.903, and 0.762, respectively.

#### Trait coping style questionnaire

This questionnaire was used to assess the coping styles of the participants. It consists of 20 items, divided into two dimensions: Positive Coping (PC) and Negative Coping (NC), each containing 10 items. Each item is scored using a 5-point Likert scale. Higher scores on a dimension indicate a stronger tendency toward the corresponding coping style (positive or negative). In this study, the overall Cronbach’s α coefficient of the questionnaire was 0.833, and the Cronbach’s α coefficients for the two dimensions were 0.882 and 0.897, respectively.

#### Turnover intention scale

This scale was developed by Michael and translated and revised by the Chinese scholar Li Dongrong (30). It consists of three dimensions: Turnover Intention I (TI I, indicating the likelihood of leaving the current job), Turnover Intention II (TI II, indicating motivation to seek other jobs), and Turnover Intention III (TI III, indicating the perceived possibility of obtaining external employment). It comprises six items using a 4-point Likert scale, where higher scores indicate stronger intent to leave. The Chinese version achieved a Cronbach’s α coefficient of 0.873 and content validity of 67.67%. In this study, the overall Cronbach’s α coefficient for the scale was 0.876, with dimension-specific Cronbach’s α coefficients ranging from 0.756 to 0.857.

### Ethical approval

This study was reviewed and approved by the Institutional Review Board of the hospital before data collection. All procedures adhered to the ethical principles outlined in the Declaration of Helsinki. Participation was entirely voluntary. Informed consent was obtained from all individual participants involved in the study. Specifically, the first page of the online questionnaire presented a detailed consent form explaining the study’s purpose and significance. Participants could only proceed to the survey questions by clicking “Agree.” The questionnaire was administered anonymously, and all responses were kept strictly confidential.

### Data analysis

Data were processed using SPSS 25.0 and AMOS 26.0 statistical software. All statistical tests were two-tailed (*α* = 0.05), with *p* < 0.05 considered statistically significant. Participants’ demographic characteristics were presented using descriptive statistics. The normality of continuous variables was assessed using the Kolmogorov–Smirnov test. Spearman correlation analysis was employed to explore the relationships among lateral violence, coping styles, psychological resilience, and turnover intention. Prior to regression analysis, continuous variables were mean-centered to mitigate multicollinearity. Hierarchical regression analysis was conducted with sociodemographic factors included as control variables to reduce their potential influence on outcome variables. AMOS version 26.0 was used for structural equation modeling (SEM) and path analysis. The hypothesized model was estimated using the maximum likelihood method. Model fit was evaluated using the following indices: *χ*^2^/df, AGFI, GFI, TLI, IFI, NFI, CFI, and RMSEA. A well-fitting model was indicated by indices meeting the recommended thresholds: *χ*^2^/df > 1.00 and <3.00, TLI > 0.90, IFI > 0.90, NFI > 0.90, CFI > 0.90, and RMSEA < 0.05. Additionally, a Bootstrap test (with 5,000 resamples) was performed to examine the significance of the indirect and total effects in the model, and the 95% bias-corrected confidence intervals are reported.

## Results

### Common method biases tests

The results of Harman’s single-factor test showed that nine factors with eigenvalues greater than 1 were extracted, with the first factor accounting for 30.466% of the total variance (<40%). This indicates no severe common method bias in this study.

### Participant characteristics

Among the 281 respondents who met the inclusion criteria, over half of the participants were female (96.1%), while males accounted for 3.9%. In terms of age, approximately 60% were under 30 years old. A total of 151 nurses were married, and 90% of the nurses held a Bachelor’s degree. The rest of the general data information is detailed in Table 1.

**Table 1:** Participants’ characteristics (*N* = 281).

Variables	Categories	*N* = 281	Percent (%)
Gender	Female	11	3.9
	Male	269	96.1
Age (years)	≤25	59	21.0
	26~	112	39.9
	31~	93	33.1
	41~	17	6.0
Marital status	Single	127	45.2
	Married	151	53.7
	Divorced/Widowed	3	1.1
Education level	Certificate	26	9.3
	Bachelor	252	89.7
	Master and above	3	1.1
Work Experience	<5	133	47.3
	5~	111	39.5
	16~	28	10.0
	30~	9	3.2
Professional Title	Junior nurse	186	66.2
	Intermediate nurse	90	32.0
	Senior nurse	5	1.8
Monthly income	<5,000	69	24.6
	5,000~	89	31.7
	8,000~	91	32.4
	11,000~	32	11.4
Employment type	Contract (temporary)	50	17.8
	Contract (long-term)	216	76.9
	Establishment	15	5.3
Department	Internal medicine	54	19.2
	Surgery	126	44.8
	Obstetrics and gynecology/Pediatrics	42	14.9
	Outpatient	3	1.1
	Emergency/ICU	53	18.9
	Others	3	1.1

### Correlations between study variables

All study variables demonstrated significant correlations. Specifically, lateral violence showed a significant negative correlation with psychological resilience (*r* = −0.319, *p* < 0.05) and positive coping (*r* = −0.272, *p* < 0.05), while exhibiting significant positive correlations with negative coping (*r* = 0.445, *p* < 0.05) and turnover intention (*r* = 0.517, *p* < 0.05). Psychological resilience was significantly positively correlated with positive coping (*r* = 0.527, *p* < 0.05) and significantly negatively correlated with negative coping (*r* = −0.444, *p* < 0.05) and turnover intention (*r* = −0.378, *p* < 0.05). Positive coping was significantly negatively correlated with turnover intention (*r* = −0.287, *p* < 0.05), whereas negative coping showed a significant positive correlation with turnover intention (*r* = 0.394, *p* < 0.05). Spearman correlation analysis are shown in Table 2.

**Table 2:** Spearman’s correlations between study variables.

Variables	Lateral violence	Psychological resilience	Positive coping style	Negative coping style	Turnover ideation
Lateral violence	1				
Psychological resilience	−0.319**	1			
Positive coping style	−0.272**	0.527**	1		
Negative coping style	0.445**	−0.444**	−0.174**	1	
Turnover ideation	0.517**	−0.378**	−0.287**	0.394**	1

***p < 0.001, **p < 0.01, *p < 0.05.

### Hierarchical linear regression results

Turnover intention scores were used as the dependent variable in the hierarchical regression analysis. Sociodemographic factors were included as control variables in the first block, lateral violence was added in the second block, psychological resilience in the third block, and both positive and negative coping styles in the fourth block (D-W = 1.988, VIF < 5, indicating no multicollinearity). The final model explained 32.8% of the variance in turnover intention (adjusted *R*^2^ = 0.328). However, in Model 4, no significant independent association was found between positive coping and turnover intention (*p* > 0.05) (Table 3).

**Table 3:** Hierarchical linear regression analysis results.

Variables	Model 1		Model 2		Model 3		Model 4	
	*β*	*t*	*β*	*t*	*β*	*t*	*β*	*t*
Gender	−0.077	−1.274	−0.006	−0.112	−0.025	−0.472	−0.057	−1.083
Age (years)	0.025	0.205	0.005	0.046	0.024	0.226	−0.024	−0.235
Marital status	0	−0.002	0.02	0.277	0.026	0.363	0.031	0.446
Education level	0.116	1.762	0.08	1.36	0.093	1.635	0.111	1.989*
Work Experience	−0.112	−1	−0.142	−1.423	−0.162	−1.677	−0.11	−1.157
Professional Title	0.006	0.065	0.022	0.272	0.023	0.293	−0.015	−0.186
Monthly income	−0.048	−0.686	0.02	0.322	0.025	0.413	0.043	0.732
Employment Type	−0.021	−0.314	−0.002	−0.027	−0.013	−0.227	−0.022	−0.384
Department	0.169	2.735**	0.12	2.171*	0.097	1.815	0.077	1.457
Lateral Violence			0.459	8.518***	0.406	7.636***	0.331	5.955***
Psychological Resilience					−0.238	−4.566***	−0.14	−2.283*
Negative Coping style							0.225	3.796***
Positive Coping style							−0.064	−1.126
*R* ^2^	0.076		0.271		0.324		0.359	
∆*R*^2^	0.045		0.244		0.296		0.328	
*F*	2.466*		10.06***		11.713***		11.51***	
Δ*F*	2.466*		72.55***		20.845***		7.352**	

***p < 0.001, **p < 0.01, *p < 0.05.

### Mediating effect analysis

**Figure 2:**
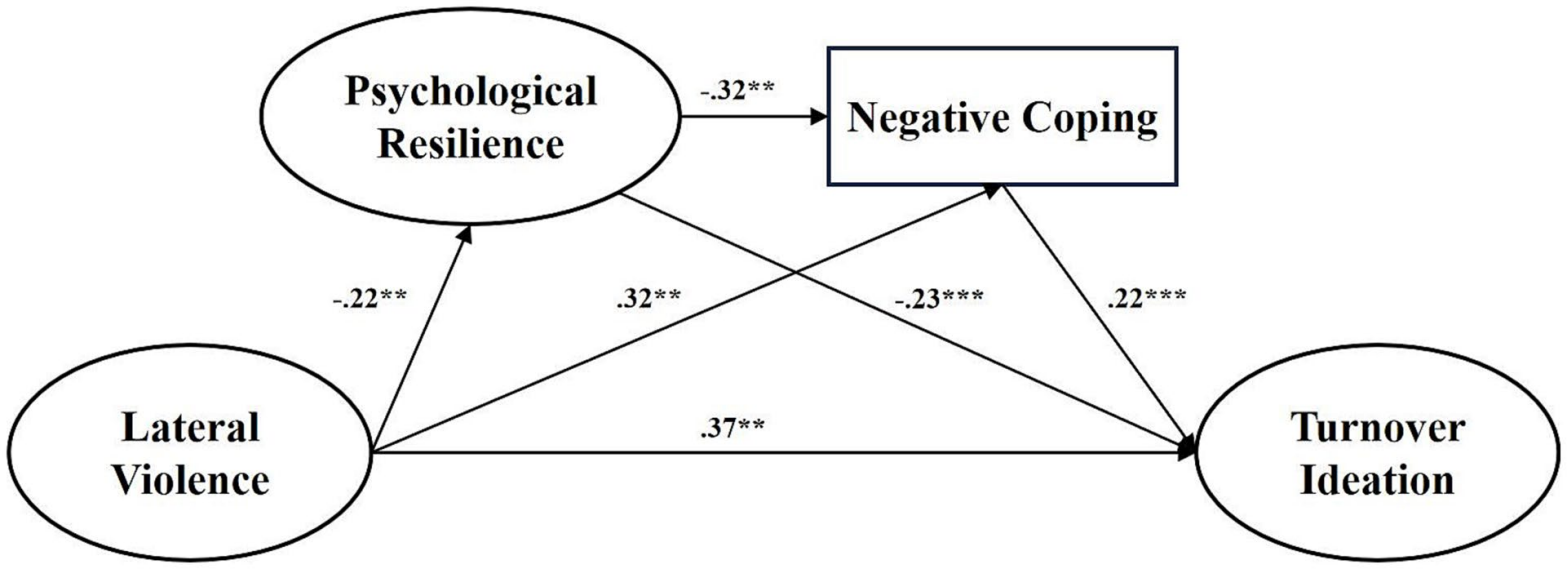
Model M0. ***p < 0.001, **p < 0.01, *p < 0.05.

The SEM analysis results indicated that the fit indices of the theoretical model were as follows: *χ*^2^ = 57.530, df = 28, *p* < 0.01, *χ*^2^/df = 2.055, AGFI = 0.926, GFI = 0.962, TLI = 0.986, IFI = 0.986, NFI = 0.973, CFI = 0.986, RMSEA = 0.061. Preliminary analysis indicated that the path from lateral violence to positive coping was not statistically significant (*p* > 0.05), and positive coping also did not show a significant effect in the hierarchical regression analysis (*p* > 0.05). Based on these statistical results and considering the specific context of this study, we decided to revise the theoretical model. In this context, the effectiveness of a positive coping style may be suppressed (an explanation that will be elaborated in detail in the Discussion section). We removed the positive coping variable and established a revised model (M0, see Figure 2). The fit indices for the M0 model were *χ*^2^ = 34.882, df = 22, *p* < 0.01, *χ*^2^/df = 1.586, AGFI = 0.947, GFI = 0.974, TLI = 0.989, IFI = 0.994, NFI = 0.983, CFI = 0.994, RMSEA = 0.046. The revised model demonstrated good fit.

**Figure 3:**
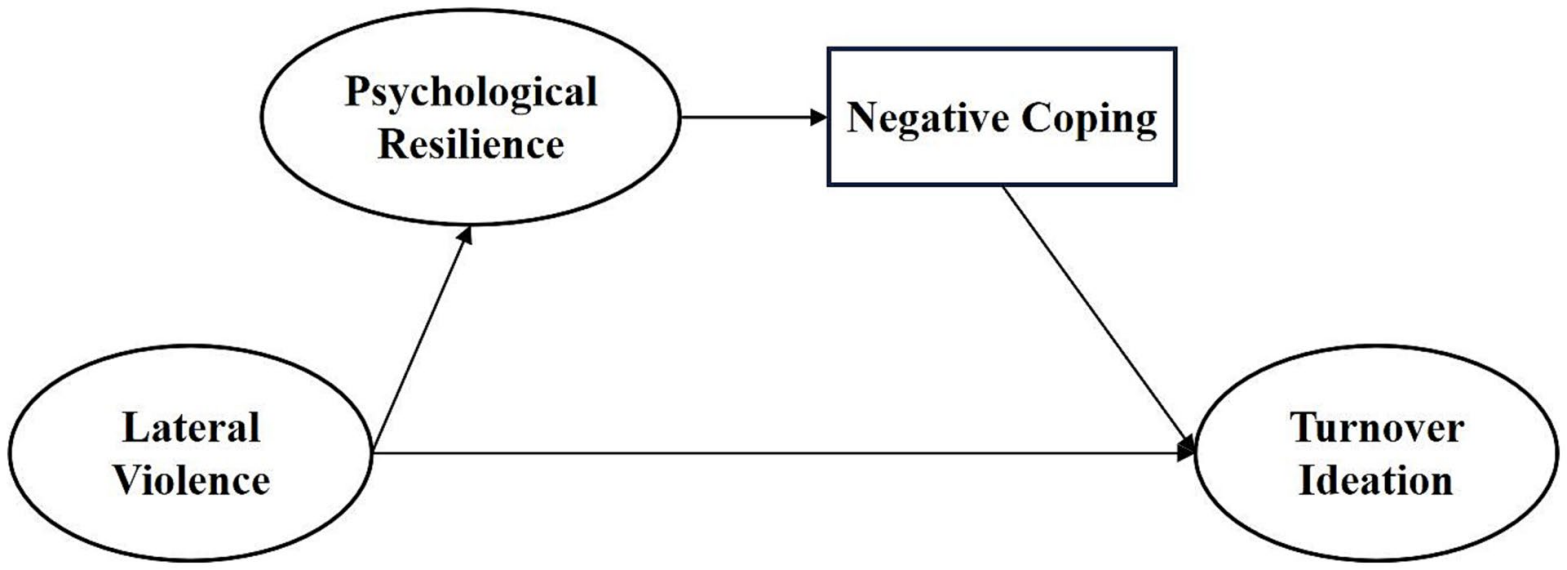
Model M1.

**Figure 4:**
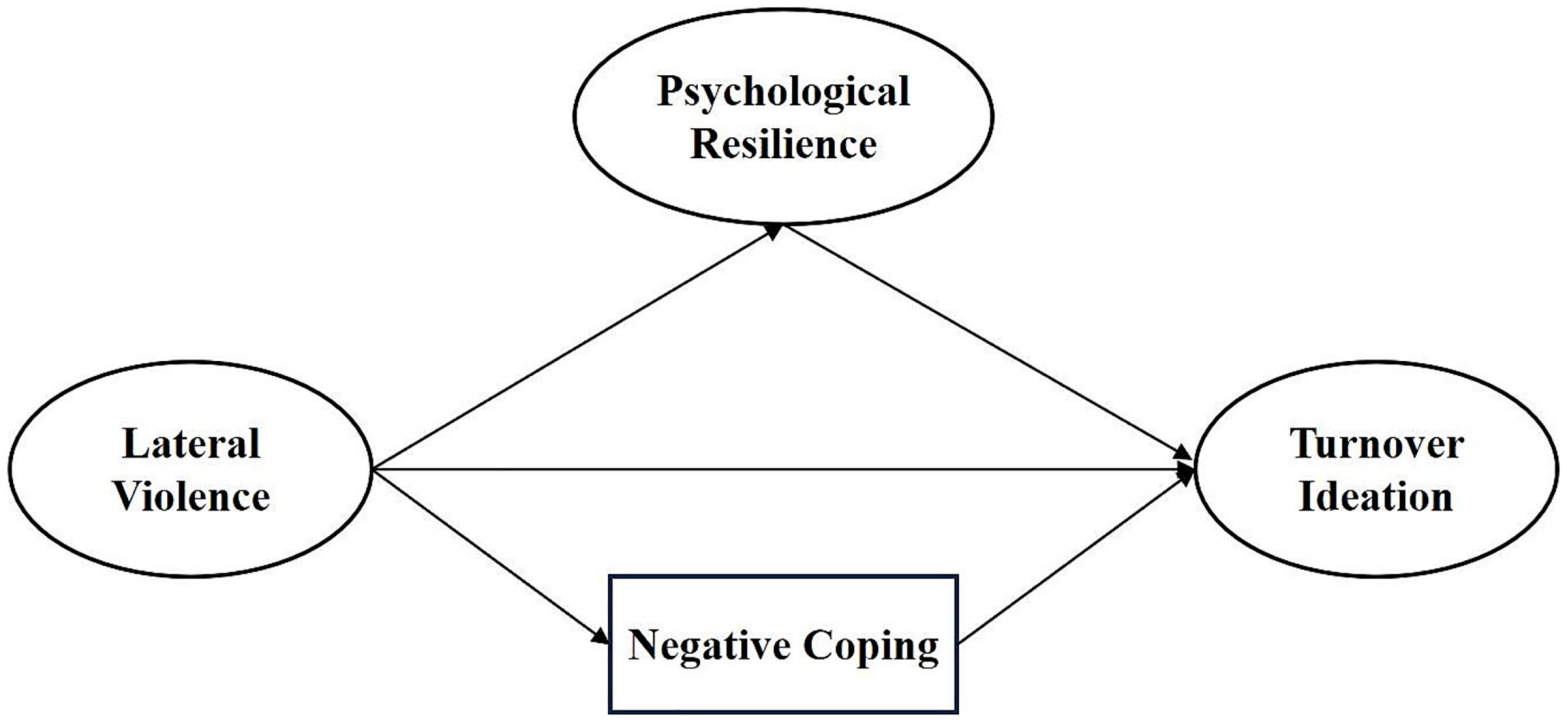
Model M2.

To test the modified model (M0) and clarify the relationship between psychological resilience and negative coping, two competing models (M1 and M2) were proposed. Competing model M1 removed the path from lateral violence to negative coping and the direct path from psychological resilience to turnover intention, forming a sequential mediation model (Figure 3). Competing model M2 removed the path from negative coping to turnover intention, resulting in a parallel mediation model (Figure 4). The fit indices for model M1 were: *χ*^2^/df = 3.462, AGFI = 0.891, GFI = 0.942, IFI = 0.971, NFI = 0.959, RMSEA = 0.094. The fit indices for model M2 were: *χ*^2^/df = 2.921, AGFI = 0.905, GFI = 0.952, IFI = 0.978, NFI = 0.967, RMSEA = 0.083. In comparison, the modified model M0 showed an adequate fit.

### Test for mediation

As shown in Table 4, all standardized direct and indirect pathways were statistically significant, confirming our theoretical hypothesis: psychological resilience and coping styles formed a concurrent and sequential chain-mediating role between lateral violence and turnover intention. Specifically, the relationship between lateral violence and turnover intention was partially mediated by psychological resilience alone (*β* = 0.052, *p* < 0.05). The relationship between lateral violence and turnover intention was also partially mediated by negative coping style alone (*β* = 0.061, *p* < 0.05). The effect of psychological resilience on turnover intention was partially mediated by negative coping styles (*β* = −0.069, *p* < 0.05). Furthermore, psychological resilience and negative coping jointly demonstrated a significant sequential mediating effect on the relationship between lateral violence and turnover intention (*β* = 0.016, *p* < 0.05).

**Table 4:** Effect estimates of the modified model.

Standardized structural paths	Direct effect (*β*)	95% CI lower, upper	Standardized Indirect effect		95% CI lower, upper	Standardized total effect
			Paths	*β*		
Lateral violence → Psychological resilience	−0.224**	(−0.380, −0.060)				−0.244**
Lateral violence→ Negative coping style	0.316**	(0.132, 0.438)	Lateral violence→ Psychological resilience→ Negative coping style	0.072**	(0.019, 0.154)	0.388**
Lateral violence→ Turnover ideation	0.365**	(0.134, 0.438)	Lateral violence→ Psychological resilience→ Turnover ideation	0.052**	(0.016, 0.113)	0.494**
			Lateral violence→ Negative coping style→ Turnover ideation	0.061***	(0.025, 0.120)	
			Lateral violence→ Psychological resilience→ Negative coping style→ Turnover ideation	0.016**	(0.004, 0.040)	
Psychological resilience→ Turnover ideation	−0.231***	(−0.347, −0.0.119)	Psychological resilience→ Negative coping style→ Turnover ideation	−0.069***	(−0.135, −0.029)	−0.301***
Negative coping style→ Turnover ideation	0.216***	(0.110, 0.334)				0.216***
Psychological resilience→ Negative coping style	−0.321**	(−0.445, −0.172)				−0.321**

***p < 0.001, **p < 0.01, *p < 0.05.

## Discussion

The study constructed a multiple mediation model between lateral violence and turnover intention and examined the chain mediating mechanism of psychological resilience and coping styles within this relationship. The analysis results indicated that the proposed theoretical model was generally supported by the data, with most research hypotheses validated. Model comparisons revealed that the revised model (excluding the positive coping variable) demonstrated better goodness-of-fit. In contrast, both competing models, M1 (containing only sequential mediation paths) and M2 (containing only parallel mediation paths), exhibited issues of inadequate fit or limited explanatory power, thereby further supporting the statistical rationale for the revised model at the statistical level. Correlation analysis showed significant associations among all variables. Lateral violence was not only directly associated with turnover intention but also exerted indirect effects through the pathways of psychological resilience and negative coping styles. Furthermore, psychological resilience and negative coping demonstrated a significant chain-mediating effect between lateral violence and turnover intention, suggesting a potential correlational sequence from stress perception to behavioral intention: lateral violence may be linked to a reduction in individuals’ psychological resilience resources, which in turn is associated with an increased use of negative coping strategies and ultimately with higher turnover intention. Specifically, nurses with lower levels of psychological resilience and a tendency to adopt negative coping strategies when experiencing lateral violence reported significantly higher turnover intentions. These findings support the core hypothesis of this study: psychological resilience and coping styles, as key psychological resources for stress response, jointly mediate the relationship between lateral violence and turnover intention. Although the American Nurses Association has issued a position statement explicitly condemning all forms of workplace violence, including bullying among colleagues, and advocating for the establishment of a zero-bullying organizational culture (31), systematic and evidence-based countermeasures in nursing management practice remain insufficient, and the failure of zero-tolerance and informational policies is well-documented (5). Against this background, this study highlights two potential intervention targets for clinical management at the level of individual psychological and behavioral mechanisms: first, enhancing nurses’ psychological resilience through targeted interventions, and second, guiding them to reduce the use of negative coping strategies.

Resilience may influence an individual’s primary appraisal of stressors, emotional metacognitive responses, and the selection of coping strategies. Through continuous interaction with their environment, individuals adjust their adaptive responses based on the nature of the challenges they face and their surrounding conditions. According to the Conservation of Resources theory, when individuals experience resource depletion, they may lower their self-expectations regarding their roles, sustain a low motivational state, and may activate defensive resource conservation strategies characterized by detachment and withdrawal. Ultimately, turnover could be viewed as an adaptive coping mechanism to terminate ongoing resource loss, a process in which the mediating role of coping styles becomes apparent. Thereby, enhancing psychological resilience is likely necessary for nurses experiencing lateral violence. Nurses with high individual resilience are generally considered better able to cope with workplace adversity, adopt constructive approaches to manage difficulties, effectively resolve interpersonal conflicts with colleagues, and thus potentially avoid emotion-focused coping styles. In contrast, nurses with lower resilience may struggle to effectively adjust their coping strategies when facing workplace violence, often showing a tendency toward avoidance, which may ultimately be linked to higher turnover intention. The findings of this study support the existing research conclusion that psychological resilience plays a mediating role between workplace violence and nurse outcomes (13, 32). Consequently, these associations suggest that psychological resilience could be a key focus in prevention and intervention strategies to mitigate the negative impacts of workplace violence and reduce turnover intention. Healthcare institutions or hospitals might consider helping nurses enhance their ability to cope with workplace violence and strengthen their professional commitment by organizing positive psychology workshops, conducting professional development programs, and equipping nurses with emotional regulation and stress management skills.

The results of this study indicate that negative coping styles play a moderating role in the relationship between lateral violence, psychological resilience, and turnover intention. This finding is consistent with previous research (33), which suggests that maladaptive coping mechanisms, such as negative coping, are associated with higher levels of job burnout and turnover intention. In fact, negative coping is a manifestation of maladjustment: while it may temporarily alleviate psychological stress in the short term, in the long run, it tends to trigger internalizing or externalizing problems, the exacerbation of chronic stress, and the continuous depletion of psychological resources, which are in turn associated with an increased risk of turnover, as it fails to address the root causes (33). Nurses who experience lateral violence often lack effective coping strategies, and this inadequacy is further correlated with turnover intention, potentially creating a vicious cycle. Currently, nursing education is increasingly recognizing the importance of coping strategies, and those affected show strong interest in learning such strategies (34). Targeted measures taken by individuals or organizations (35, 36), such as problem-solving training and mindfulness interventions, may help nurses develop more positive coping styles, which could alleviate their psychological burden and be linked to a reduction in turnover intention caused by workplace bullying. It should be noted that in an environment with insufficient organizational support, overemphasizing positive coping and personal responsibility may be counterproductive. Therefore, it could be beneficial to strengthen problem-centered coping training and incorporate it into systematic intervention and development programs with the aim of mitigating the negative impact of bullying and stabilizing the nursing workforce. On this basis, some scholars recommend moving interventions earlier, introducing structured progressive courses during nursing education that systematically cover different types of bullying, their consequences, prevention, and coping strategies. This approach aims to foster a collaborative and supportive professional culture from the outset of their careers, which might help in breaking the cycle of “Nurses Eat Their Young” and contributing to a healthy, sustainable practice environment (20, 37).

Surprisingly, our study did not find a significant mediating effect of positive coping. This result differs from previous research that suggests nurses tend to favor positive coping strategies (38, 39). In model testing, positive coping showed no statistically significant effect, whereas negative coping demonstrated stronger predictive power and a more pronounced mediating role. This discrepancy may be because when an individual’s coping strategies do not align with their actual ability to alter the stressor, merely relying on positive and constructive coping efforts may yield limited benefits. This also reflects the dynamic nature of coping styles across different contexts. Previous studies similarly indicate that nurses subjected to workplace bullying often adopt more passive, emotion-focused coping approaches (40, 41). This may mirror their real-life experiences and adaptive processes in the face of bullying, where sustaining a positive response can be difficult when the environment cannot be effectively changed. Moreover, research shows that in the absence of adequate organizational culture and supportive environments, positive coping strategies may fail to alleviate stress effectively and could even negatively impact nurses’ professional quality of life, highlighting the need for a comprehensive understanding and appropriate recognition of the role of negative coping (42). Therefore, at the organizational level, nursing managers should focus on fostering an inclusive and supportive professional culture. This includes encouraging nurses to share their experiences of lateral violence and related emotional distress in a safe and trustworthy environment while providing clear channels for communication and support (35). Given that transforming organizational culture requires considerable time and that interventions must be closely tailored to the situation of those affected by bullying, it is recommended to prioritize the development and implementation of systematic support plans centered on safeguarding nurses’ mental health and professional well-being. Examples include strengthening caring leadership practices and establishing responsive organizational support mechanisms to address the issue. Relevant studies have found that authentic leadership plays a moderating role in relational conflicts (43). Notably, existing studies have confirmed that a caring leadership style can reduce the risk of nurses experiencing negative workplace behaviors (44). Providing organizational support to nurses who have been bullied can not only enhance their job satisfaction but also effectively lower their turnover intention (45).

Internationally, multiple strategies are being promoted to address lateral violence, aiming to establish a zero-tolerance organizational and cultural atmosphere and implement prevention at the institutional level. In reality, eliminating such negative behaviors in healthcare requires commitment from all nurses. Both individuals and organizations need to take responsibility for any uncivil conduct. It is a professional duty for nurses to demonstrate mutual care and respect and integrate acts of kindness into their professional practice. Crucially, without sustained oversight, consistent policy enforcement, and proactive measures, any efforts are unlikely to yield effective results. This study identified lateral violence, psychological resilience, and coping styles as factors associated with nurses’ turnover intention. Notably, beyond their independent effects, psychological resilience and negative coping styles together form a significant chain-mediating pathway between lateral violence and turnover intention. These finding highlights potential targets for intervention. Effective interventions could be designed that aim not only to reduce lateral violence itself but also to bolster individuals’ psychological resources and promote adaptive coping strategies. Consequently, future research and practice should develop integrated prevention and intervention strategies targeting the individual, organizational, and systemic levels. Nursing managers can identify nurses with lower psychological resilience and a preference for negative coping strategies through screening and preventive measures, and implement targeted interventions to help them improve their psychological resilience, cope with stress constructively, and effectively mitigate the negative impacts of lateral violence, reducing turnover risk.

### Limitations

The following factors need to be considered. First, reliance on self-reported questionnaires for data collection carried a possibility of recall bias; combining semi-structured interviews could yielded more comprehensive results. Second, the use of Harman’s single-factor test as a preliminary screening tool cannot fully rule out the possibility of common method bias, and the test itself is sensitive to model specification. Additionally, the cross-sectional design limits the ability to draw causal inferences between the examined variables. Therefore, longitudinal studies are warranted to further explore these relationships. Finally, convenience sampling restricted the representativeness of the sample; multicenter and cross-regional studies would have better elucidated the developmental mechanisms of turnover intention among nursing staff.

## Conclusion

Nurses’ experience of lateral violence is closely associated with their psychological resilience, coping styles, and turnover intention. Psychological resilience and coping styles were identified as significant mediators in the relationship between lateral violence and turnover intention among nurses. These results provide evidence for the correlations among lateral violence, psychological resilience, coping styles, and turnover intention, and may offer insights for the development and implementation of workplace cultural safety systems for nursing staff.

## Data Availability

The original contributions presented in the study are included in the article/supplementary material, further inquiries can be directed to the corresponding author.
